# Novel coronavirus pneumonia and acute pulmonary thromboembolism: casualty or causality?

**DOI:** 10.31744/einstein_journal/2020AI5750

**Published:** 2020-05-29

**Authors:** Gabriel Laverdi Beraldo, Eduardo Kaiser Ururahy Nunes Fonseca, Patrícia Yokoo, Marina Justi Rosa de Matos, Marcela Emer Egypto Rosa, Murilo Marques Almeida Silva, Rodrigo Caruso Chate, Hamilton Shoji, Walther Yoshiharu Ishikawa

**Affiliations:** 1 Hospital Israelita Albert Einstein, São PauloSP Brazil Hospital Israelita Albert Einstein, São Paulo , SP , Brazil .

A great concern in the current global scenario, the pandemic caused by the novel coronavirus (COVID-19) has been one of the main reasons for chest computed tomography requests, both in early evaluation or late control. COVID-19 patients present an increased D-dimer, a finding likely related to hypercoagulability status and marker of worse prognosis. ^( 1 )^ We report two cases with laboratory confirmation of coronavirus and acute pulmonary embolism – an association already described. ^( 2 , 3 )^

**Figure 1:**
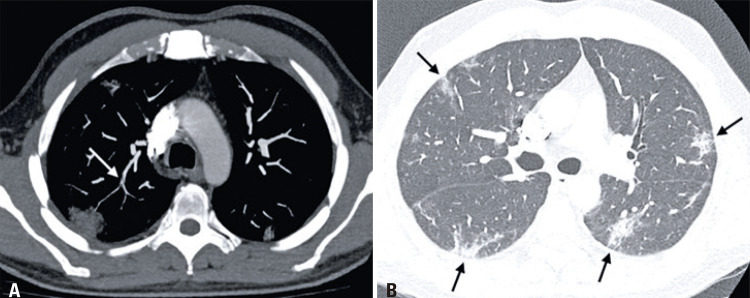
Computed tomography pulmonary angiography (49-year-old patient). (A) Axial mediastinal window shows filling defects of segmental and subsegmental artery branches of the right superior lobe (white arrows), findings compatible with pulmonary embolism. (B) Axial pulmonary window reveals multiple ground-glass opacities and thickened interlobular septae with bilateral and peripheral distribution (black arrows), findings consistent with coronavirus pneumonia

A 49-year-old man who underwent a computed tomography pulmonary angiography (CTPA) due to a worsening of dyspnea, newly onset of chest pain and D-dimer elevation (711ng/dL) on the seventh day of hospitalization. A filling defect was observed on the segmental artery branch in the right superior lobe, a compatible finding with pulmonary embolism ( Figure 1A ), as well as typical changes caused by coronavirus pneumonia ( Figure 1B ). ^( 4 , 5 )^

**Figure 2:**
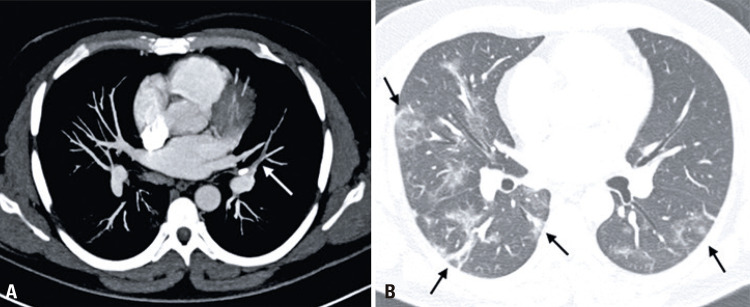
Computed tomography pulmonary angiography (41-year-old patient). (A) Axial mediastinal window shows lingular segmental pulmonary embolism (white arrows). (B) Axial pulmonary window reveals multiple ground-glass opacities, thickened interlobular septae, and parenchymal bands with bilateral and mainly peripherally distribution, findings consistent with COVID-19 pneumonia

A 41-year-old man with worsening of dyspnea and D-dimer elevation (1.427ng/dL) on the sixth day of hospitalization. A CTPA was performed, confirming pulmonary embolism on the lingular segmental branch ( Figure 2A ), in addition to the typical findings of COVID-19 pneumonia ( Figure 2B ). ^( 4 , 5 )^

Similar to other inflammatory processes, the COVID-19 pneumonia causes endothelial dysfunction and increases pro-coagulation activity. ^( 6 )^ The elevation of D-dimer due to the infection is a finding that marks poor prognosis (higher hospitalization and death rates). ^( 1 )^ There are reports of other cases of pulmonary embolism among these patients, however, this association is still unclear. ^( 2 , 3 )^ We must be alert to pulmonary embolism in patients infected by COVID-19, particularly in cases of sudden worsening of dyspnea, given that overlap of clinical and laboratory findings makes the correct diagnosis more difficult, impacting on the morbidity and mortality.
