# Supplementary material for: Novel coronavirus pneumonia and acute pulmonary thromboembolism: casualty or causality?
Source: Einstein (Sao Paulo). 2020 May 29;18:eAI5750. doi: 10.31744/einstein_journal/2020AI5750 (PMC7259829; doi:10.31744/einstein_journal/2020AI5750)
Supplement: Translation

# Translation: Pneumonia pelo novo coronavírus e tromboembolismo pulmonar agudo: casualidade ou causalidade?

**Authors:** Gabriel Laverdi Beraldo, Eduardo Kaiser Ururahy Nunes Fonseca, Patrícia Yokoo, Marina Justi Rosa de Matos, Marcela Emer Egypto Rosa, Murilo Marques Almeida Silva, Rodrigo Caruso Chate, Hamilton Shoji, Walther Yoshiharu Ishikawa

**Affiliations:** 1 Brasil Hospital Israelita Albert Einstein, São Paulo, SP, Brasil .

Motivo de grande preocupação no cenário global atual, a pandemia pelo novo coronavírus (COVID-19) tem sido um dos grandes motivos da realização de tomografias computadorizadas de tórax, seja na avaliação inicial ou no controle evolutivo. Esses pacientes apresentam aumento do dímero D, achado provavelmente relacionado a um estado de hipercoagulabilidade e que marca pior prognóstico.^( 1 )^A seguir, ilustramos dois casos de pacientes com confirmação laboratorial de infecção pelo coronavírus e sinais de tromboembolismo pulmonar (TEP) – associação já descrita.^( 2 , 3 )^

Paciente do sexo masculino, 49 anos, encaminhado para angiotomografia do tórax devido à piora da dispneia, surgimento de dor torácica e elevação do dímero D (711ng/dL) no sétimo dia de internação. Evidenciou-se falha de enchimento em ramo arterial segmentar do lobo superior direito, compatível com TEP ( Figura 1A ), bem como alterações típicas para a pneumonia pelo coronavírus ( Figura 1B ).^( 4 , 5 )^

**Figura 1:** Angiotomografia computadorzada de tórax (paciente de 49 anos). (A) Corte axial em janela de mediastino evidenciando falhas de enchimento de ramos arteriais segmentares e subsegmentares no lobo superior direito (seta branca), achados compatíveis com tromboembolismo pulmonar. (B) Corte axial em janela para avaliação do parênquima pulmonar com múltiplas opacidades em vidro fosco, associadas a espessamento de septos interlobulares, com distribuição bilateral e periférica (setas pretas), achados consistentes com pneumonia por coronavírus

Paciente do sexo masculino, 41 anos, evolui com piora da dispneia e elevação do dímero D (1.427ng/dL) no sexto dia de internação. Submetido à angiotomografia do tórax, que confirmou sinais de TEP em ramo segmentar lingular ( Figura 2A ), além dos achados típicos da pneumonia pelo coronavírus ( Figura 2B ).^( 4 , 5 )^

**Figura 2:** Angiotomografia computadorzada de tórax (paciente de 41 anos). (A) Corte axial em janela de mediastino evidenciando tromboembolismo pulmonar em ramo arterial segmentar lingular (seta branca). (B) Corte axial em janela para avaliação do parênquima pulmonar revela múltiplas opacidades em vidro fosco com espessamento de septos interlobulares, por vezes associadas a bandas parenquimatosas, achados bilaterais e predominantemente periféricos, consistentes com pneumonia por coronavírus

Assim como outros processos inflamatórios, a pneumonia pela COVID-19 determina disfunção endotelial e aumento da atividade pró-coagulativa.^( 6 )^A elevação do dímero D em vigência da infecção é um achado observado e que se relaciona com pior desfecho (maiores taxas de internação e óbito).^( 1 )^Há relatos de outros casos de TEP nesses pacientes, ainda com associação incerta.^( 2 , 3 )^Devemos estar atentos para a suspeita TEP em pacientes infectados pelo novo coronavírus, principalmente em casos de piora abrupta de dispneia e do padrão respiratório, uma vez que a sobreposição de achados clínicos e laboratoriais dificulta o correto diagnóstico e impacta na morbimortalidade.
